# CD8^+^ T-Cell Responses to Trypanosoma cruzi Are Highly Focused on Strain-Variant *trans*-Sialidase Epitopes

**DOI:** 10.1371/journal.ppat.0020077

**Published:** 2006-08-04

**Authors:** Diana L Martin, D. Brent Weatherly, Susana A Laucella, Melissa A Cabinian, Matthew T Crim, Susan Sullivan, Mark Heiges, Sarah H Craven, Charles S Rosenberg, Matthew H Collins, Alessandro Sette, Miriam Postan, Rick L Tarleton

**Affiliations:** 1 Center for Tropical and Emerging Global Diseases, University of Georgia, Athens, Georgia, United States of America; 2 Instituto Nacional de Parasiologia Dr. Mario Fatala Chaben/Administración Nacional de Laboratorios e Institutos de Salud “Dr. Carlos G. Malbrán” (ANLIS/Malbran), Buenos Aires, Argentina; 3 Department of Microbiology, University of Georgia, Athens, Georgia, United States of America; 4 Department of Cellular Biology, University of Georgia, Athens, Georgia, United States of America; 5 La Jolla Institute for Allergy and Immunology, San Diego, California, United States of America; National Institutes of Health, United States of America

## Abstract

CD8^+^ T cells are crucial for control of a number of medically important protozoan parasites, including *Trypanosoma cruzi,* the agent of human Chagas disease*.* Yet, in contrast to the wealth of information from viral and bacterial infections, little is known about the antigen specificity or the general development of effector and memory T-cell responses in hosts infected with protozoans. In this study we report on a wide-scale screen for the dominant parasite peptides recognized by CD8^+^ T cells in T. cruzi–infected mice and humans. This analysis demonstrates that in both hosts the CD8^+^ T-cell response is highly focused on epitopes encoded by members of the large *trans*-sialidase family of genes. Responses to a restricted set of immunodominant peptides were especially pronounced in T. cruzi–infected mice, with more than 30% of the CD8^+^ T-cell response at the peak of infection specific for two major groups of *trans*-sialidase peptides. Experimental models also demonstrated that the dominance patterns vary depending on the infective strain of *T. cruzi,* suggesting that immune evasion may be occurring at a population rather than single-parasite level.

## Synopsis

The authors of this paper conducted a broad screen to identify the major proteins in *Trypanosoma cruzi,* the causative agent of Chagas disease, that allow for detection and control of this intracellular pathogen by CD8^+^ T cells. This study is the first to show that a complex pathogen such as T. cruzi elicits a T-cell response focused on a few peptides, despite having a genome of >12,000 genes capable of encoding hundreds of thousands of potential target epitopes. The immunodominant CD8^+^ T-cell targets in both murine and human T. cruzi infection are almost exclusively peptides within multiple *trans*-sialidase proteins that are encoded by the large and diverse *trans*-sialidase gene family. *trans*-sialidase genes show great potential for variation, and the frequency of individual *trans*-sialidase epitopes appears to vary significantly in different parasite strains, giving rise to distinct patterns of T-cell responses to different T. cruzi isolates. The authors hypothesize that the massive expansion of this gene family under immunological pressure and the resulting variable expression of specific T-cell epitopes provides a mechanism of immune escape for *T. cruzi.*

## Introduction

CD8^+^ T-cell responses participate in immune control of a wide range of intracellular pathogens, including viral, bacterial, and protozoal pathogens [1]. Antigen (Ag)–specific CD8^+^ T-cell responses in viral and bacterial infections are focused on a small number of epitopes [2–4], and examination of these Ag-specific T cells ex vivo has enhanced our understanding of pathogen-specific immune responses and has provided information needed for more rational vaccine design [5–8]. The study of immune responses to protozoan parasites provides a notable contrast, with a dearth of information on the epitope specificity of parasite-specific CD8^+^ T cells and thus little understanding of the targets of controlling and protective immune responses. Diseases caused by intracellular protozoan parasites contribute significantly to morbidity and mortality worldwide, both as vector-borne and opportunistic infections. *Plasmodium* spp, *Toxoplasma gondii, Leishmania major,* and Trypanosoma cruzi all induce strong CD8^+^ T-cell responses that are crucial for control of these infections [9,10–14]. In addition, the vaccine-induced generation of CD8^+^ T-cell responses results in protective immunity in experimental infection models [12,15–17]. However, the inability to detect Ag-specific CD8^+^ T cells directly ex vivo has hindered progress in understanding the development and maintenance of parasite-specific CD8^+^ T-cell responses against parasitic pathogens.

A number of potential factors might account for the lack of identified immunodominant CD8^+^ T-cell epitopes in parasitic infections. Immunodomination is dictated by several factors, including the abundance of source protein, peptide generation in the proteasome, peptide affinity for major histocompatibility complex (MHC) in the case of T-cell responses, and composition of the T-cell receptor repertoire [18,19]. It is possible that the large size of the genomes and the complexity of the proteomes of these parasites preclude the focusing of CD8^+^ T-cell responses to a few immunodominant epitopes. The T. cruzi genome, for example, encodes more than 12,000 genes, providing thousands of potential epitopes that presumably compete for presentation by class I MHC on the surface of *T.cruzi*–infected host cells.

Previous studies have identified a few T. cruzi peptides, including some encoded by the *trans*-sialidase (ts) family of genes, as targets for cytolytic T cells in mice and in humans infected with T. cruzi [20–25]. However, responses to these previously studied epitopes appear to be of low frequency and there are no published reports of the direct ex vivo detection of T cells specific for any of these epitopes. It is possible that previously described epitopes are in fact only minor targets of CD8^+^ T-cell responses in T. cruzi–infected hosts, and that bona fide dominant epitopes have yet to be identified. However, the lack of described immunodominant epitopes, not only from T. cruzi but from any protozoan parasite, raises the question of whether the repertoire of potential targets is so vast in complex eukaryotic pathogens that no individual or set of epitopes would dominate the CD8^+^ T-cell response. The studies described here investigate these two possibilities by expanding the list of potential epitopes and reveal that the CD8^+^ T-cell response to T. cruzi in both mice and humans is highly focused on a small set of epitopes primarily encoded by genes of the large and strain-variant ts gene family.

## Results

### ts Epitopes Dominate the CD8^+^ T-Cell Response in T. cruzi–Infected Mice

We first expanded the list of potential ts epitopes by scanning a set of raw sequencing reads that were made available prior to the release of the T. cruzi genome for peptides predicted to bind to murine H-2K^b^ using known epitopes as templates (see Materials and Methods). This analysis yielded a total of 404 peptides with sequence similarity to the three previously described H-2K^b^–binding epitopes (VDYNFTIV (pep77.2), VNHRFTLV (PA8), and VNHDFTVV (PA14) [21]) from among the 43,000 selected raw sequence reads with homology to known ts genes. An alignment of published ts genes with the reads containing these peptides revealed 324 predicted peptides that were potentially present in the same relative position in the source ts genes as the three template epitopes (Figure S1). Of these 324 peptides, 156 are present in the 1,430 ts genes in the annotated T. cruzi genome (77 of these are present only in the 695 ts pseudogenes). The predicted H-2K^b^ binding potential was determined computationally for each of these peptides, and the combination of frequency of representation in the genome and predicted MHC binding efficiency (Table S1) was then used to prioritize peptides for further screening.

**Figure 1:**
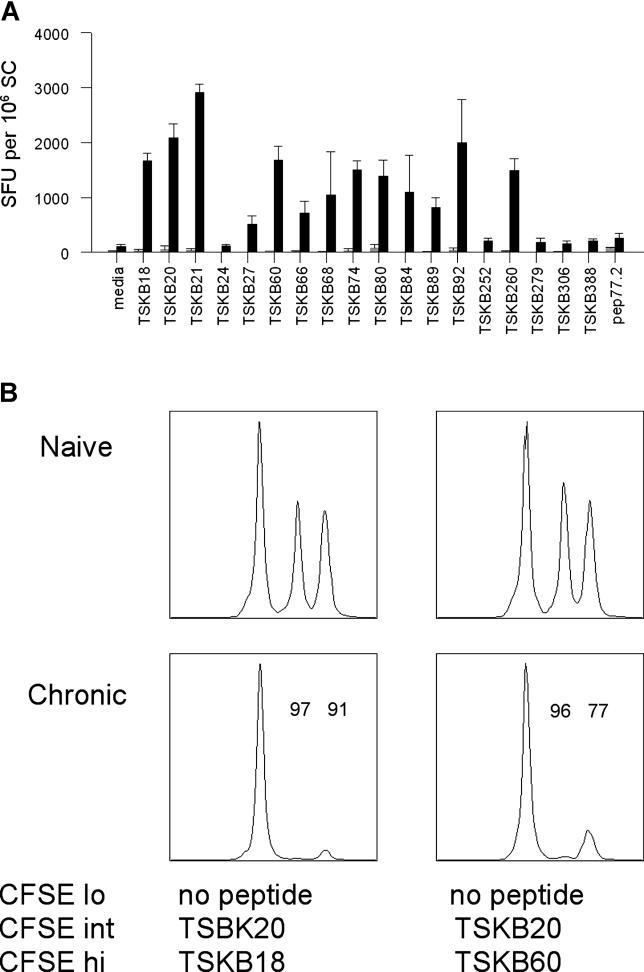
Functional Responses of Murine CD8^+^ T Cells Directed against T. cruzi–Derived ts Peptides (A) SCs from naive (gray bars) or chronically infected (Brazil strain; black bars) B6 mice were stimulated overnight with ts peptides on anti-IFNγ–coated ELISPOT plates as described in Materials and Methods. Error bars represent SD. Data represent the number of SFUs per 10^6^ SCs plated. (B) SCs from naive B6 mice were loaded with T. cruzi peptides and labeled with CFSE as described in Materials and Methods. Cells were recovered from naive and infected mice 16 h after transfer and examined for CFSE fluorescence. The numbers above the peaks represent the percentage of specific killing for targets loaded with the respective peptide, and was calculated as described in Materials and Methods. Data are representative of eight experiments.

Approximately 100 of the predicted H-2K^b^–binding ts peptides were tested individually or in pools for their ability to induce IFNγ production from splenocytes of chronically infected mice. Fourteen ts peptides induced IFNγ elaboration by spleen cells (SCs) directly ex vivo (Figure 1A); however, five of these (TSKB24, TSKB27, TSKB66, TSKB68, and TSKB84) were recognized by T cells from fewer than 50% of mice tested (Table S1) and were not considered further. Of the remaining nine epitopes, seven were also found to be targets for cytolytic T cells as determined by in vivo elimination of peptide-loaded SCs (Figure 1B, and Table S1). These results confirmed that peptide-responsive CD8^+^ T cells with a full range of functional activity are maintained in mice during the chronic phase of T. cruzi infection.

**Figure 2:**
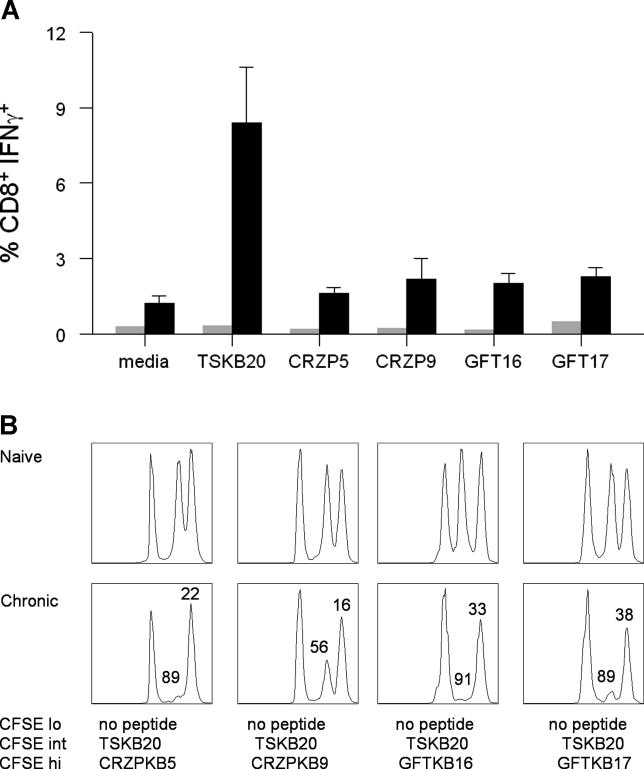
Functional Responses of Murine CD8^+^ T Cells Directed against Non-ts Epitopes (A) SCs from naive (gray bars) or chronically infected (Brazil strain; black bars) B6 mice were stimulated for 5 h with TSKB20, cruzipain, or GFT peptides plus GolgiPlug as described in Materials and Methods. Data represent the number of CD8^+^ T cells producing IFNγ; error bars represent SD. (B) SCs from naive B6 mice were loaded with T. cruzi peptides and labeled with CFSE as described in Materials and Methods. The numbers above the peaks represent the percentage of specific killing of the target cells loaded with the indicated peptide and was calculated as described in Materials and Methods. Data are representative of three experiments.

In addition to members of the ts family, several other proteins have been shown to be targets of CD8^+^ T cells following T. cruzi infection using classical restimulation/killing assays [23,26]. To expand the coverage of potential CD8^+^ T-cell targets beyond the ts family, we also analyzed predicted H-2K^b^–binding peptides encoded by other genes and gene families. Among these are peptides found within cruzipains (CRZPs), mucin-associated surface proteins (MASPs), β-galactofuranosyl transferase (GFTs), and gp63 proteins (Table S2). A number of criteria were used to select these peptides for evaluation as potential targets of CD8^+^ T cells, including factors that make a protein available for processing through the MHC I pathway, such as the predicted presence of a signal sequence and/or glycosylphosphatidyl inositol anchor addition site on the protein [27], detection of the protein in amastigotes or trypomastigotes of T. cruzi as determined by whole proteome analysis [28], and the presence of the peptides in multiple members of a gene family (Table S2). Of the 93 peptides tested by IFNγ enzyme-linked immunospot (ELISPOT) analysis, four (CRZP5, CRZP9, GFT16, and GFT17) were found to elicit T-cell responses from mice with chronic Brazil strain infections (Figure 2A). These non-ts epitopes induced IFNγ from only 40%–60% of mice tested and did so only in overnight, but not short-term (5-h) cultures. Frequencies of IFNγ-producing cells were consistently 4- to 20-fold lower against non-ts epitopes compared to ts epitopes in both acutely and chronically infected mice (unpublished data). Target cells loaded with non-ts epitopes were also poorly recognized in in vivo cytotoxic T lymphocyte assays in acutely or chronically infected mice (unpublished data and Figure 2B). These results indicate that these non-ts peptides generate low frequency T-cell responses following Brazil strain T. cruzi infection, further supporting the conclusion that the epitope specificity of CD8^+^ T-cell responses in experimental T. cruzi infection is strongly dominated by ts gene family–encoded peptides.

**Figure 3:**
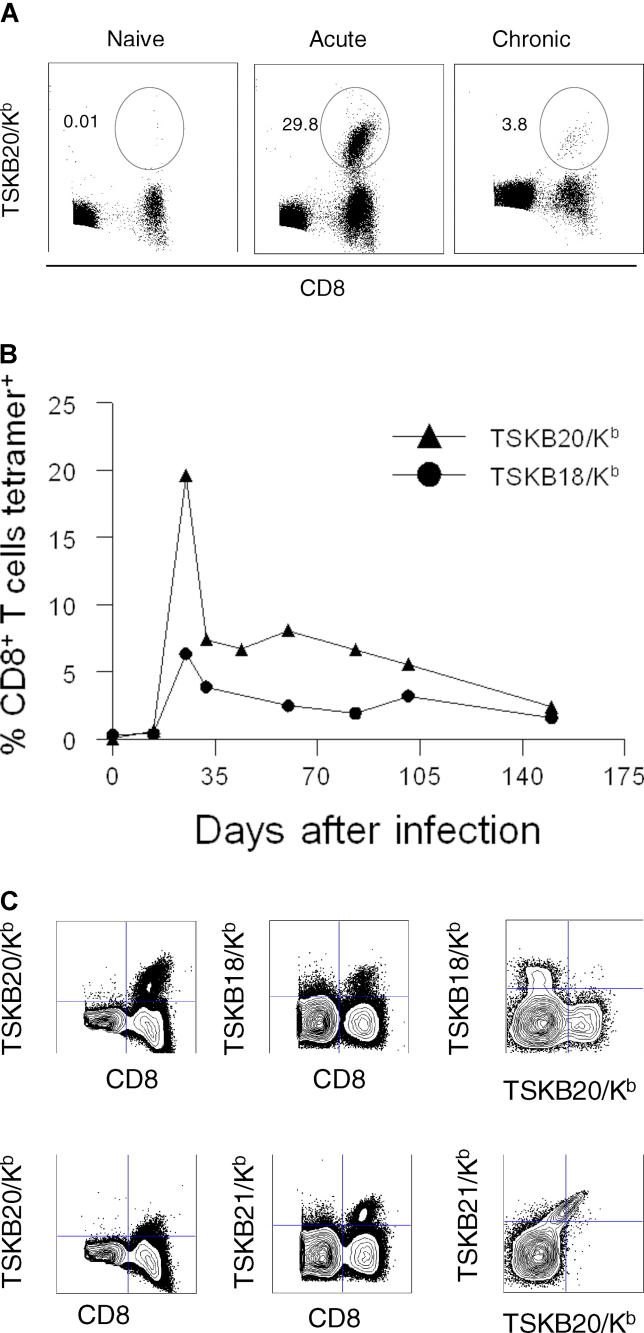
Magnitude, Kinetics, and Cross-Reactivity of CD8^+^ T-Cell Responses to Dominant ts Epitopes (A) SCs from naive or chronically infected (Brazil) B6 mice were stained with TSKB20/K^b^-PE tetramers, then anti-CD8 APCs and a Cy5PE exclusion channel consisting of anti-CD4, anti-CD11b, and anti-B220. Cells shown are gated on Cy5PE^neg^lymphocyte^+^ populations. Numbers represent the percentage of CD8^+^ T cells staining with the TSKB20/K^b^ tetramer. (B) Kinetics of TSKB20 (closed triangles)– and TSKB18 (open triangles)–specific responses during Brazil strain infection of B6 mice. Data are representative of three experiments (*n* = 5 mice per group). (C) SCs from infected mice were stained with anti-CD8–FITC, TSKB20/K^b^-PE, anti-CD4/11b/B220 cocktail, and either TSKB18/K^b^–APC (top) or TSKB21/K^b^–APC (bottom). Left panel shows CD8 versus TSKB20/K^b^; middle panel shows CD8 versus TSKB18/K^b^ or TSKB21/K^b^; right panel shows TSKB18/K^b^ or TSKB21/K^b^ tetramer staining versus TSKB20/K^b^ staining, gated on CD8^+^ lymphocytes.

To further explore the magnitude and kinetics of ts-specific CD8^+^ T-cell responses during experimental infection, MHC I tetramers bearing two of these epitopes, TSKB18 and TSKB20, were tested for direct ex vivo binding to CD8^+^ T cells from Brazil strain T. cruzi–infected mice. The TSKB20-specific CD8^+^ T-cell response shows significant expansion, reaching a peak of nearly 30% of all CD8^+^ T cells at 21 days after infection, followed by contraction and stabilization at 2%–5% of CD8^+^ T cells in chronically infected mice (Figure 3A and 3B). The kinetics of the TSKB18-specific T-cell response follows a similar pattern, but the overall level is consistently 1.5- to 4-fold lower than for TSKB20-specific T cells throughout the course of infection (Figure 3B). The massive TSKB20-specific T-cell response demonstrates that the CD8^+^ T-cell response to T. cruzi can be focused to a very few epitopes even within the context of tens of thousands of potential targets and among the hundreds of other similar ts epitopes that are present in this complex parasite.

Because of the sequence similarity between the dominant ts epitopes shown to be the targets of CD8^+^ T-cell responses in murine T. cruzi infection, we next determined whether these peptides were recognized by unique or overlapping populations of CD8^+^ T cells. TSKB20/K^b^ and TSKB18/K^b^ tetramers clearly bind discrete populations of Ag-specific CD8^+^ T cells (Figure 3C, top right), whereas TSKB20/K^b^ and TSKB21/K^b^ tetramers bind to the same population of T cells (Figure 3C, bottom right). Costaining with TSKB21/K^b^ reduces the intensity of TSKB20/K^b^ fluorescence, whereas costaining with TSKB18/K^b^ does not (Figure 3C, left panel), indicating competition between TSKB21/K^b^ and TSKB20/K^b^ for binding to the same T-cell receptor. TSKB260, which differs from TSKB20 only at the P8 K^b^ anchor residue, also cross-reacts with the TSKB20-specific T-cell population (unpublished data). (For simplicity, the population of T cells responding to TSKB20, TSKB21, and TSKB260 is referred to as the “TSKB20-specific T-cell response.”) Likewise, TSKB18, TSKB74, TSKB80, and TSKB89 are all recognized by the same T-cell population as determined by tetramer analysis and peptide-induced IFNγ production from TSKB18-specific T cells (unpublished data). Therefore, peptides contained within ts proteins generate both distinct and overlapping/cross-reactive immunodominant CD8^+^ T-cell populations.

### CD8^+^ T-Cell Responses in T. cruzi–Infected Human Subjects Are Also Directed against a Few ts Epitopes

**Figure 4:**
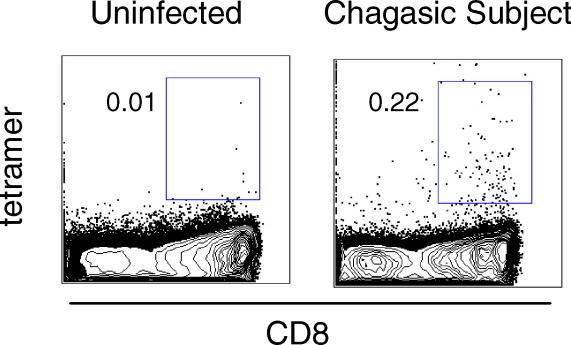
Detection of CD8^+^ T Lymphocytes Recognizing HLA-A2.1–Restricted ts Epitopes in Patients with Chronic Chagas Disease PBMCs were stained using MHC tetramers specific for the TSA2–38 or TSA2–44 epitopes. The percentage of CD8^+^ T cells staining positive for TSA2–38 is shown for a representative chronic chagasic patient and an uninfected control. Cells shown are lymphocytes gated on CD4^neg^B220^neg^CD11b^neg^ lymphocytes. Five out of eight IFNγ ELISPOT^+^ subjects stained positive with tetramers.

To determine whether peptides from ts proteins are also major targets of CD8^+^ T cells in humans chronically infected with *T. cruzi,* a similar approach to that described above for the selection of predicted H-2K^b^–binding peptides was used to identify 71 peptides with sequence homology to previously characterized HLA-A2–binding ts epitopes [25]. The initial screen for HLA-A2 binding and T-cell recognition used SCs from HLA-A2.1 transgenic mice with chronic T. cruzi infections as responders in ex vivo, IFNγ ELISPOT assays. Thirty-two of the 71 peptides reproducibly induced IFNγ production (Figure S2), and were further tested using peripheral blood mononuclear cells (PBMCs) from 17 HLA-A2.1^+^ chronic chagasic subjects. IFNγ-secreting cells were observed in ten (59%) of 17 subjects, with 24 of the 28 peptides assayed being recognized by at least one subject (range, two to seven subjects/peptide; Table S3). Four of the seven nonresponding subjects also failed to recognize T. cruzi Ags derived from an amastigote lysate preparation, confirming the poor CD8^+^ T-cell responses in some of these subjects as previously reported [26]. Although immunodominance of particular peptides was not as obvious as that observed in mice, T cells from each of the responding subjects recognized either or both Ts38 and Ts44 peptides. MHC class I tetramers containing peptides Ts38 and Ts44 also detected a low frequency of parasite-specific CD8^+^ T cells (% CD8^+^ tetramer^+^ = 0.15–0.40) in five of eight chronic chagasic patients who had positive IFNγ ELISPOT responses for either one or both peptides (Figure 4). These peptides are the only reported epitopes recognized directly ex vivo by PBMCs from T. cruzi–infected humans, demonstrating that ts peptides are the predominant targets of CD8^+^ T cells in both mice and humans.

### Parasite Strain Diversity Significantly Influences the Magnitude of Ag-Specific Responses and Patterns of Immunodominance

Although ts epitopes appear to be dominant in both mouse and human T. cruzi infection, the difference in magnitude of the anti-ts CD8^+^ T-cell response in B6 mice and humans is striking. Two of the differences in experimental infection of mice and natural infection in humans are the diversity of infective parasite strains and polymorphic MHC molecules in naturally infected hosts. Increasing MHC I diversity in experimental models minimally affects the magnitude and hierarchy of the dominant TSKB20-specific T-cell response as determined by Brazil strain infection of F1 crosses of B6 mice with CBA (B6CBAF1/J, H-2^b/k^) and Balb/c (CB6F1/J, H-2^b/d^) mice (Figure S3)*.*

**Figure 5:**
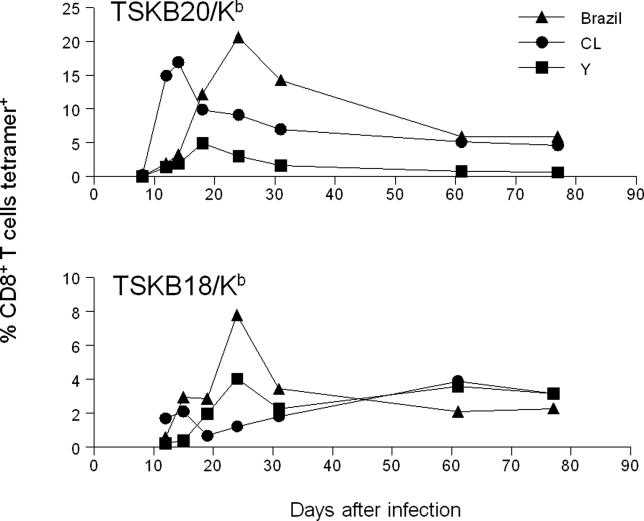
Parasite Strain–Dependent Dominance Patterns in T. cruzi–Infected Mice B6 mice were infected with 1,000 Brazil, 1,000 Y, or 10,000 CL strain T. cruzi and stained with TSKB20/K^b^ (top) or TSKB18/K^b^ (bottom) tetramers as described in Materials and Methods. Data shown are a combination of two separate experiments with three or four mice per group per experiment.

To address the question of whether the infecting strain of parasite influences the magnitude of CD8^+^ T-cell responses to the dominant T-cell epitopes TSKB20 and TSKB18, we infected mice with Brazil, Y, or CL strains of T. cruzi and followed the peptide-specific T-cell responses over time. Surprisingly, mice infected with different strains of T. cruzi generated CD8^+^ T-cell responses differing in magnitude, kinetics, and patterns of dominance (Figure 5). Brazil strain–infected mice generated higher frequencies of both TSKB20- and TSKB18-specific CD8^+^ T cells than CL strain– or Y strain–infected mice (Figure 5). TSKB20-specific T-cell responses peaked earliest following CL infection (Figure 5, top), although these frequencies were slightly lower than those observed following Brazil strain infection. In stark contrast, TSKB20-specific T-cell responses in mice infected with Y strain parasites were 4- to 6-fold lower than those generated following CL or Brazil strain infection (Figure 5, top). In mice infected with any of the given strains of *T. cruzi,* the TSKB20-specific T-cell response peaks in the acute phase, then contracts and remains at relatively constant levels throughout the course of chronic infection (Figure 5, top), as is typical of dominant CD8^+^ T-cell responses in most viral and bacterial infections [1]. T-cell responses to TSKB18 following CL or Y strain infection displayed somewhat delayed and considerably sustained (>30 d) expansion compared to TSKB20-specific T-cell responses. Three patterns of dominance subsequently emerge in chronically infected mice: TSKB20 > TSKB18 (Brazil), TSKB20 ≈ TSKB18 (CL), and TSKB20 < TSKB18 (Y). These data document that immunodominance in CD8^+^ T-cell responses to T. cruzi varies considerably depending on the infecting strain of parasites, perhaps reflecting differences in expressed ts genes in different strains.

Evidence for significant variation in ts epitope expression among T. cruzi strains can be found in data from the sequencing of the T. cruzi genome. The CL Brener clone (from the CL strain) was the primary source for the T. cruzi genome sequencing [29]. However ~2.5× coverage of the Esmeraldo strain, an apparent relative of one of the parental strains of the CL Brener hybrid, was also completed and allows us to compare the representation of predicted K^b^-binding epitopes in these 2 strains; a sample of the results of this comparison is shown in Table 1. In the most extreme case, TSKB364 is greater than 25 times more prevalent in the Esmeraldo sequence reads than those from the CL Brener strain, while one of the immunodominant epitopes, TSKB18, is present in approximately twice the number of relative sequence reads in CL Brener than in Esmeraldo. In addition, a number of the predicted epitopes are present in the reads of only one strain but not the other. Therefore, the immunodominant targets of the CD8^+^ T-cell response in T. cruzi are also potentially highly variable among parasite strains.

**Table 1:**
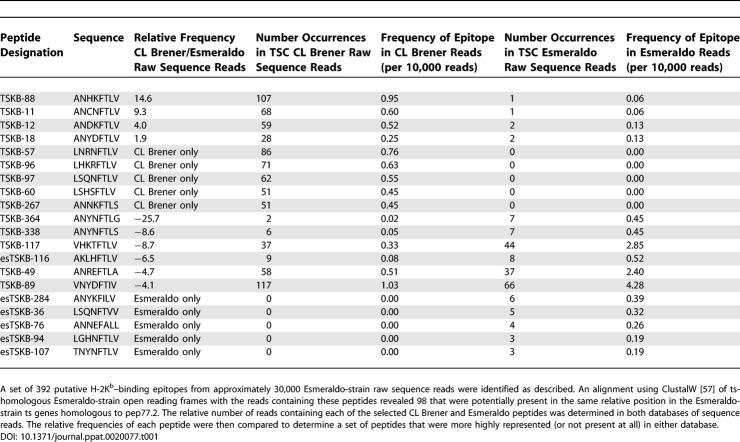
Differential Distribution of ts Genes among Closely Related T. cruzi Strains

## Discussion

Herein we demonstrate that CD8^+^ T-cell responses in T. cruzi infection are tightly focused on a small subset of peptides encoded by the ts gene family. Two of these peptides, TSKB20 and TSKB21, are recognized by an overlapping set of CD8^+^ T cells that at the peak of the response accounts for greater than 30% of the total circulating CD8^+^ T-cell pool in B6 mice infected with the Brazil strain of *T. cruzi.* Typical reported frequencies of pathogen epitope-specific CD8^+^ T cells detected directly ex vivo in the spleen or blood of mice infected with various viral or bacterial agents are approximately 2%–20% of all CD8+ T cells [30–33]. Thus, the TSKB20-specific CD8^+^ T-cell response in T. cruzi infection described herein is among the strongest documented responses against a single epitope in any infectious disease. Lymphocytic choriomeningitis virus represents a notable exception: more than 50% of CD8^+^ T cells in infected BALB/c mice recognize the NP_118–126_ epitope [34]. The kinetics of ts epitope–specific responses is quite distinct from other well-studied infection models. The time to peak response (19–24 d) following Brazil strain infection mirrors the rise and peak of parasite load in this model, suggesting that the stimulating epitopes are limiting early in the infection. Not unexpectedly, the ts-specific response in the B6/Brazil model contracts as the infection is controlled and continues a slow decline in the postacute infection period, again reflecting the chronic nature of this infection. In contrast, in most viral infections exhibiting strong immunodominant CD8 responses, peak viremia and CD8^+^ T-cell responses occur within 1 wk after infection [35].

The massive response to TSKB20 was unexpected given the overall complexity of the T. cruzi genome, whose more than 12,000 genes includes more than 700 ts family members and nearly 700 ts pseudogenes. In addition, the ts family is only one of several families encoding thousands of surface-membrane and secreted glycoproteins that would be possible targets of CD8^+^ T-cell responses in this infection [28,29,36,37]. Despite this complexity, the remarkable dominance of the response by T cells specific for a few ts peptides is established early and largely remains for the length of the infection, confirming that immunodominance is an inherent property of CD8^+^ T-cell responses, irrespective of the epitope complexity of pathogens. Likewise, T. cruzi–infected Balb/c mice also generate significant ts-specific CD8^+^ T-cell responses against the H-2K^d^ epitope IYNVGQVSI (Turrentine et.al., unpublished data; and [38]). Identification of these potent immunodominant CD8^+^ T-cell responses also firmly establishes that immunity to T. cruzi is not dominated by immunosuppression nor by nonspecific polyclonal responses, as has been previously proposed [39,40].

**Figure 6:**
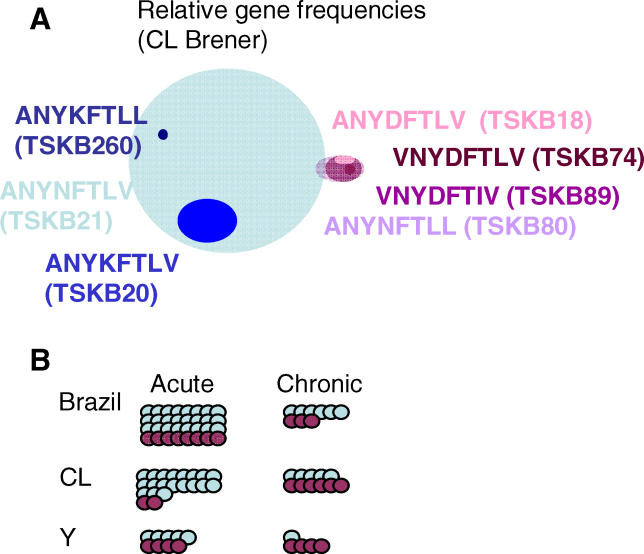
Relative Frequencies of Epitope Occurrences in the Genome and CD8^+^ T-Cell Responses (A) Venn diagram representing relative frequencies and overlap of T cells responding to T. cruzi epitopes. The blue shaded circles (left) show the high-abundance overlapping peptides TSKB21 and TSKB20, along with the low-abundance overlapping peptide TSKB260. These peptides are among the most highly represented H-2K^b^–binding peptides in the proteome and elicit the strongest CD8^+^ T-cell response following infection with Brazil strain *T. cruzi,* as shown in (B). The smaller purple shaded circles (right) show the lower-abundance epitopes TSKB18, TSKB74, TSKB80, and TSKB89, which are recognized by T-cell populations that overlap minimally with TSKB20/21/260-specific T cells. (B) Relative frequencies of CD8^+^ T cells recognizing the TSKB20 (blue) and TSKB18 (purple) families of peptides in the acute (left) and chronic (right) phase of T. cruzi infection in mice infected with three different strains. While there are strain-dependent differences in the magnitude of the TSKB20-specific T-cell responses, these responses all follow a similar pattern of expansion and contraction during acute infection, which may be explained simply by strain-dependent differences in the rates of parasite replication and/or expression levels of TSKB20. However, T-cell responses to TSKB18 vary remarkably in both their magnitude and kinetics following infection with the various strains. Sustained expansion of TSKB18-specific T cells following infection with CL or Y strains results in different hierarchies of Ag-specific CD8^+^ T cells in chronic infection with the three different strains (right).

A number of factors, including peptide affinity for MHC I, peptide liberation by cellular proteases, abundance of the source gene product, the CD8^+^ T-cell repertoire, and the kinetics of expression of the determinant [18,19], have been proposed to contribute to immunodominance, but epitope abundance appears to be a key determinant [41]. In retrospect, the dominance of the TSKB20/21 response in T. cruzi infection is predictable, as these epitopes are encoded by a combined 229 genes in the CL Brener strain genome, 90 of which also have evidence of expression in the Brazil strain proteome, by far the highest among the H-2K^b^–binding ts peptides examined (Table S1). A combination of MHC I tetramer staining (Figure 3) and assays of T-cell receptor downregulation indicate that the peptides comprising the TSKB20 group are full agonists for T cells recognizing any of the peptides in the group, but fail to significantly interact with T cells specific for peptides in the TSKB18 group (Figure 6 and unpublished data). Immunodominance by the TSKB18 group of peptides is less easily predicted based on gene frequency, although collectively this set of epitopes is encoded in a total of 18 genes, seven of which were detected by proteome analysis. However, the true expression levels of these epitopes in infected cells is difficult, if not impossible, to determine. Gene frequency is also clearly not a perfect predictor of epitope abundance and possible recognition by T. cruzi–specific CD8^+^ T cells; other higher frequency, potential K^b^-binding ts-encoded peptides (e.g., TSKB4) fail to generate a detectable CD8^+^ T-cell response, and K^b^-binding peptides encoded in hundreds of MASP genes were also not recognized by CD8^+^ T cells from infected mice (Table S2).

ts proteins are major surface and released proteins in extracellular trypomastigotes and in intracellular amastigotes of T. cruzi and are also primary targets of humoral immune responses. The enzymatically active ts proteins are responsible for a number of crucial activities in trypomastigotes; paramount among these is the transfer of sialic acid from host proteins to T. cruzi glycoproteins, a process that helps provide resistance to complement-mediated lysis and assists in host cell invasion by these extracellular forms [42–45]. However, the vast majority of ts genes encode proteins that lack enzymatic activity [46], and the function of this set of ts proteins is not known, nor is the biological role of ts molecules expressed by intracellular amastigotes of T. cruzi clear. Comparative analysis of kinetoplastid genomes suggests that there has been a dramatic expansion of the ts genes in T. cruzi but not in closely related kinetoplastids such as T. brucei and L. major [47]. It is plausible that host immune responses have been the major selective force for the expansion of the ts family in *T. cruzi,* given that the ts proteins are the dominant targets of both humoral and cellular immune responses in T. cruzi–infected hosts, and the absence of other explanations for the presence of hundreds of enzymatically inactive ts family proteins. The location of many of the ts genes in subtelomeric regions of chromosomes, where recombination and the formation of new alleles is facilitated, is noteworthy, as this is also the locale for large families of genes involved in immune evasion in other protozoans [48].

If immune pressure has provided the evolutionary force for the explosive diversification of the ts genes, then what is the selective advantage for parasites to express many of these ts family members, particularly since certain epitopes appear to be potent immune targets [15,22,38]? Simultaneous expression of hundreds of ts genes could prevent the focusing of the immune response by the sheer volume of distinct peptides presented to the immune system. However, this is clearly not the case with respect to the H-2K^b^–binding epitopes identified in this study. The large number of closely related epitopes encoded by ts genes may also cross-antagonize each other via altered peptide ligand effects [49]. We have demonstrated the ability of several ts peptides to antagonize T cells specific for one ts epitope (Cabinian et al., unpublished data), and we show herein that ts epitopes can serve as full or partial agonists for T cells responsive to other ts epitopes, indicating a degree of promiscuity in T cells recognizing ts peptides.

A third potential advantage that a large and potentially highly variable repertoire of ts genes may provide for T. cruzi is a reservoir of proteins that may be expressed at different points in the infection or disease, or in different strains or isolates. Previous studies [28,50] demonstrate that, collectively, T. cruzi isolates simultaneously express many ts proteins, but it is not known if the expressed ts repertoire varies between individual parasites in a host, or within the parasite population in an individual host over the course of infection. However, genome comparisons provide support for a substantial variation in the ts repertoire in distinct T. cruzi isolates; exact copies of ts genes documented in GenBank from various T. cruzi strains are not present in the CL Brener strain genome and the presence and frequency of individual ts peptides varies considerably between the CL Brener and Esmeraldo strains (Table 1). We propose that it is this variation that accounts for the distinct patterns of epitope specificity and evolution of the ts-specific CD8^+^ T-cell response in mice infected with different parasite strains (Figure 5). Strain variance in ts genes may also account for lower and more diffuse patterns of ts-specific responses in human subjects, although exhaustion of the T-cell response over decades of the infection in these subjects is also a likely factor [51].

Therefore, although the full flexibility for creation and expression of different ts family proteins in individual parasites, between parasite strains, and over the course of infection is not known, the potential for this variation clearly exists and may be exploited by T. cruzi to help evade immune responses. These possibilities should be considered in deciding whether ts molecules are good vaccine candidates because they are immunodominant, or poor candidates because they are also highly variable among strains and perhaps over time. Likewise, it is also important to remember that despite the strong dominant T-cell responses to ts epitopes in C57BL/6 mice, these mice fail to clear the infection but remain persistently infected and go on to develop symptoms of chronic disease [52].

This report provides the first description in any parasitic infection of epitopes that allow for the direct ex vivo identification of parasite-specific T cells over the course of the infection. Like numerous other protozoan and metazoan parasites, T. cruzi provides unique challenges to research due to strain diversity, the large genomes, the often chronic nature of the infection and, particularly in the case of *T. cruzi,* the overwhelming number and diversity of families of related genes that are immunological targets. The ability to detect and track Ag-specific T cells will be crucial for following the initiation and maintenance of T. cruzi–specific CD8^+^ T-cell responses in infected hosts and should help provide insights into the mechanisms of parasite persistence and disease pathology in this important human disease.

## Materials and Methods

### Mice and parasites.

C57BL/6 (B6) mice were bred in our animal facility in microisolator cages under specific pathogen–free conditions. B6CBAF1/J and CB6F1/J mice were obtained from Jackson Laboratories (Bar Harbor, Maine, United States). HLA-A2 transgenic mice were bred from pairs obtained from Dr. L. Sherman through Harlan Sprague-Dawley, Inc. (Indianapolis, Indiana, United States). Tissue culture trypomastigotes of the Brazil strain of T. cruzi were obtained from passage through Vero cells. Mice were infected intraperitoneally with 1,000 Brazil strain tissue culture trypomastigotes and were killed by CO_2_ inhalation. In some experiments mice were infected intraperitoneally with 1,000 Y or 10,000 CL parasites obtained through passage in Vero cells or mice. All animal protocols were approved by the University of Georgia Institutional Animal Care and Use Committee.

### Selection of study population.

HLA-A2.1^+^ T. cruzi–infected adult volunteers were recruited at the Chagas disease Section of the Cardiology Department, Hospital Interzonal General de Agudos “Eva Perón,” Buenos Aires, Argentina [26]. T. cruzi infection was determined by indirect immunofluorescence assay, hemagglutination, and enzyme-linked immunosorbent assay techniques performed in the Clinical Laboratory of Parasitology in the Hospital Interzonal General de Agudos “Eva Perón.” This study was approved by the Institutional Review Boards of the University of Georgia, and the Hospital Interzonal General de Agudos “Eva Perón.”

### Peptides.

H-2K^b^– and HLA-A2.1–binding peptides from ts genes were predicted using sequence homology searching of available data (GenBank,  http://www.ncbi.nlm.nih.gov/Genbank), and raw T. cruzi genome sequencing reads (courtesy of the T. cruzi Genome Sequencing Consortium [TSC]) for variants of the known H-2K^b^–binding peptides pep77.2, PA8, and PA14 [21], and selected HLA-A2.1–binding peptides [25]. Initially, 404 and 845 potential variants of the H-2K^b^– and HLA-A2.1–binding peptides, respectively, were identified. A similar analysis of T. cruzi Esmeraldo strain raw sequence reads yielded an additional 98 homologous, potential H-2K^b^–binding ts epitopes. Non-ts peptides were determined using H-2K^b^–motif predictions of selected genes in the TSC annotated genome as described previously [53]. Peptides were synthesized by the University of Georgia Molecular Genetics Instrumentation Facility (Athens, Georgia, United States), Pepscan Systems (Lelystad, the Netherlands), or SigmaGenosys (Saint Louis, Missouri, United States).

### Lymphocyte culture.

Single-cell suspensions of splenocytes from uninfected or T. cruzi–infected mice were generated using previously published methods [54]. For patient studies, approximately 50 ml blood from patients and control subjects was drawn by venipuncture into heparinized tubes (Vacutainer; Becton-Dickinson, San Jose, California, United States). PBMCs were isolated by density gradient centrifugation as previously described [26].

### HLA-A2.1 typing.

HLA-A2.1 typing was performed as previously described [25] by incubating 1 × 10^6^ PBMCs with HLA-A2.1–specific mAb BB7.2 [55] (10 μg/ml; American Type Culture Collection, Bethesda, Maryland, United States) for 30 min at 4 °C, followed by FITC-labeled F(ab′)_2_ goat anti-mouse IgG (1/50 dilution; Immunotech, Marseille, France). Positive fluorescence indicating expression of HLA-A2.1 was assessed by analysis of cells using fluorescence microscopy (Nikon, Tokyo, Japan).

### ELISPOT.

ELISPOT assays to measure secretion of IFNγ from peptide-stimulated mouse splenocytes or human PBMCs were performed as per manufacturer's instructions (BD Pharmingen, San Diego California, United States), and spots were counted using an Immunospot analyzer (CTL, Cleveland, Ohio, United States).

### Intracellular cytokine staining.

SCs from naive or infected mice were stimulated at 37 °C with 5% CO_2_ for 5 h with 5 μM peptide in the presence of GolgiPlug (BD Pharmingen) to block cytokine export. Cells were permeabilized and stained with anti-CD8 FITC and anti-IFNγ APCs as previously described [54]. At least 150,000 cells were acquired on a BD FacsCalibur flow cytometer (BD Pharmingen) then analyzed with FlowJo (Tree Star, Ashland, Oregon, United States) using a biexponential transform.

### In vivo cytotoxicity assay.

In vivo cytotolytic activity was assessed as previously described [54,56]. Spleen cells from naive mice were either incubated with individual T. cruzi peptides or with no peptide for 1 h at 37 °C. Cells were washed twice with PBS, then incubated with CFSE (Molecular Probes, Eugene OR) (2 μM = CFSE^hi^, 0.4 μM = CFSE^int^, 0.1 μM = CFSE^lo^) as described in the legends to Figures 1B and 2B. After quenching with FCS, cells were combined and transferred intravenously to naive and chronically infected mice. After 16 h, spleens were harvested and CFSE-labeled cells were detected using a Cyan flow cytometer (Cytomation, Fort Collins, Colorado, United States). Percentage of specific killing was determined using the formula: 1− [(% CFSE^lo^ naive / % CFSE^int/hi^ naive) / (% CFSE^lo^ chronic / % CFSE^int/hi^ chronic)] × 100%.

### MHC I tetramer staining.

MHC I tetramers were synthesized at the Tetramer Core Facility (Emory University, Atlanta, Georgia, United States). Tetramers used in these studies were: TSKB18 (ANYDFTLV/K^b^), TSKB20 (ANYKFTLV/K^b^), TSKB21 (ANYNFTLV/K^b^), TSA2–38 (FANHKFTLV/A2), and TSA2–44 (FANYKFTLV/A2). In kinetics experiments, mouse peripheral blood was obtained by retro-orbital venipuncture and collected in Na citrate solution. In other experiments, human PBMCs or mouse splenocytes were analyzed. Single-cell suspensions of splenocytes or PBMCs were washed in PAB, stained for 15 min at 37 °C with tetramers, then stained 30 min at 4 °C with labeled anti-CD8 antibodies as well as anti-CD4, anti-CD11b, and anti-B220 for use as an exclusion channel. For mouse kinetics experiments, whole blood was costained with TSKB20/K^b^ PE and TSKB18/K^b^ allophycocyanin tetramers, stained for surface markers, then lysed in a hypotonic ammonium chloride solution. At least 500,000 cells were acquired on a BD FacsCalibur flow cytometer (BD Pharmingen) then analyzed with FlowJo (Tree Star) using a biexponential transform.

### Statistical analysis.

Statistical significance in murine experiments was calculated using a two-tailed student *t* test.

## Supporting Information

Figure S1 All 735 Annotated ts Genes in the TSC T. cruzi Genome Were Aligned Using ClustalW The portion of the alignment containing the peptides of interest from a subset of the aligned sequences are shown, with the original template peptides highlighted in green and the template-homologous peptides in yellow. In addition, the designation assigned to the peptide and the locus tag (taken from http://TcruziDB.org) of a gene containing it are shown on the left side. Note: some peptides are contained in many different genes, and only a subset of those genes are included in the figure (e.g., TSKB21 is contained in 198 annotated ts genes, but only three are shown).
(2.0 MB TIF)

Figure S2 IFNγ Responses of HLA-A2 Transgenic Mice to ts Peptides HLA-A2 transgenic mice were infected with 1,000 Brazil strain tissue culture trypomastigotes. After 150 d, SCs were stimulated with various ts peptides in an ELISPOT assay. Data are displayed as spot-forming units (SFUs) per 150,000 spleen cells; error bars represent SD.
(168 KB PDF)

Figure S3 Increasing MHC I Diversity Does Not Alter TSKB20-Specific T-Cell Frequencies in T. cruzi–Infected Mice B6, B6xCBA (B6CBAF1/J), or B6xBalb/c (CB6F1/J) mice were infected with 1,000 Brazil strain trypomastigotes, then serially bled and examined for TSKB20-specific T cells by MHC I tetramer staining. Each point is the average of five mice per group and represents two separate experiments. Asterisks denote the two timepoints (D18 and D21 after infection) at which B6CBAF1/J mice had significantly lower (*p* = 0.007 and 0.01, respectively) frequencies of TSKB20-specific CD8^+^ T cells than parental B6 mice.
(55 KB PDF)

Table S1 Summary of Genomic, Proteomic, and Immunologic Data for ts Peptides Predicted to Bind H-2K^b^
(160 KB XLS)

Table S2 Summary of Genomic, Proteomic, and Immunologic Data for Non-ts Peptides Predicted to Bind H-2K^b^
(52 KB XLS)

Table S3 Summary of Genomic, Proteomic, and Immunologic Data for ts Peptides Predicted to Bind HLA-A2 (33 KB XLS)

## Abbreviations

Ag: antigen
ELISPOT: enzyme-linked immunospot
MHC: major histocompatibility complex
PBMC: peripheral blood mononuclear cell
SC: spleen cell
ts: trans-sialidase
TSC: T. cruzi Genome Sequencing Consortium
