# Supplementary material for: CD8+ T-Cell Responses to Trypanosoma cruzi Are Highly Focused on Strain-Variant trans-Sialidase Epitopes
Source: PLoS Pathog. 2006 Aug 4;2(8):e77. doi: 10.1371/journal.ppat.0020077 (PMC1526708; doi:10.1371/journal.ppat.0020077)
Supplement: Supplementary file 1 [file ppat.0020077.sg002.pdf]

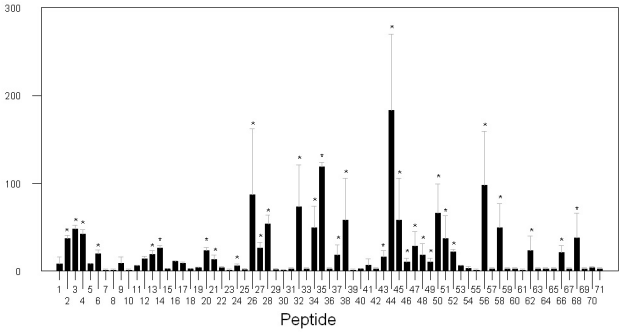

300
200
100
0
SFU/1.5x10⁵ PBMC
Peptide
