# Supplementary material for: CD8+ T-Cell Responses to Trypanosoma cruzi Are Highly Focused on Strain-Variant trans-Sialidase Epitopes
Source: PLoS Pathog. 2006 Aug 4;2(8):e77. doi: 10.1371/journal.ppat.0020077 (PMC1526708; doi:10.1371/journal.ppat.0020077)
Supplement: Supplementary file 2 [file ppat.0020077.sg003.pdf]

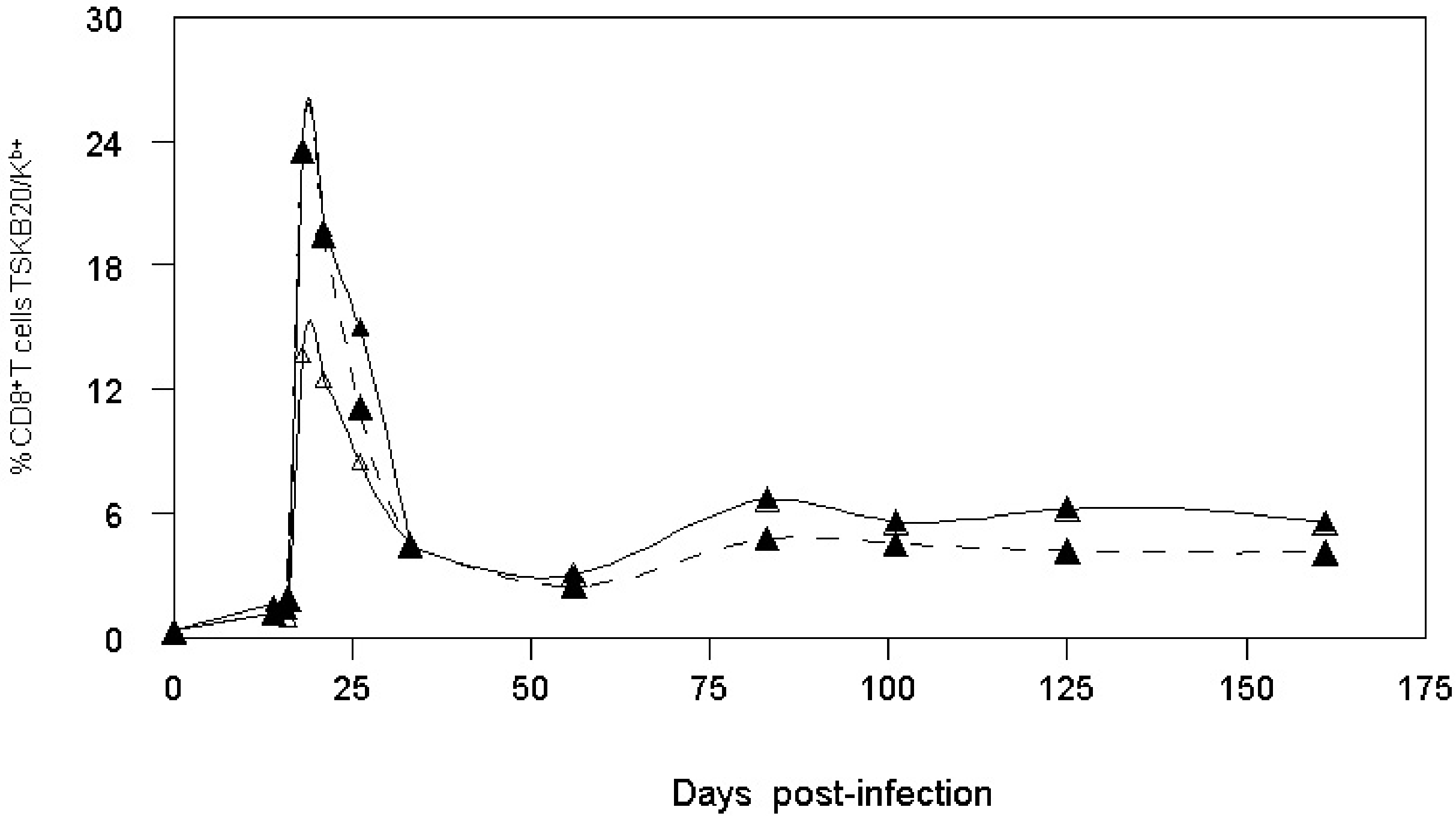

% CD8+ T cells TSKB20/Kb+
30
24
18
12
6
0
0
25
50
75
100
125
150
175
Days post-infection
